# The Effects of High-Dose Qinggan Huoxue Recipe on Acute Liver Failure Induced by D-Galactosamine in Rats

**DOI:** 10.1155/2013/905715

**Published:** 2013-03-11

**Authors:** Hong Zhu, Yang Zhang, Xiaoyu Hu, Cheng Yi, Sen Zhong, Yanyan Wang, Fang Yang

**Affiliations:** ^1^Department of Abdominal Cancer, West China Hospital, Sichuan University, Chengdu 610041, Sichuan, China; ^2^Department of Infectious Diseases, Affiliated Hospital of Chengdu University of Traditional Chinese Medicine, Chengdu 610072, Sichuan, China; ^3^Department of Clinical Medicine, Chengdu University of Traditional Chinese Medicine, Chengdu 610072, Sichuan, China

## Abstract

Qinggan Huoxue Recipe is a traditional Chinese medicine, which has been usually used to improve liver function in hepatitis. In order to investigate the effects of high-dose Qinggan Huoxue Recipe on acute liver failure and explore the potential mechanism, we had built acute liver failure models in rats by intraperitoneal injection of D-galactosamine (D-GalN). High-dose Qinggan Huoxue Recipe was delivered by gavage. After treatment, the blood alanine aminotransferase (ALT), aspartate aminotransferase (AST), total bilirubin (TBIL), albumin (ALB), cholinesterase (CHE), and prothrombin time (PT) were determined. The pathological score of liver tissue was recorded. Proliferating cell nuclear antigen (PCNA) immunohistochemistry staining and fluorescence quantitative reverse transcription polymerase chain reaction (qRT-PCR) of high mobility group box 1 (HMGB1), toll-like receptor 4 (TLR4), nuclear factor-kappa B (NF-**κ**B), and Caspase-3 were performed. The survival curve was also depicted. Our results demonstrated that high-dose Qinggan Huoxue Recipe could significantly improve liver function and increase survival rates in rats with acute liver failure. These effects were supposed to be mediated by suppressing inflammatory reaction and apoptosis.

## 1. Introduction

Acute liver failure (ALF) is a life-threatening medical emergency and occurs when the liver rapidly loses its function within a short period. ALF can develop secondary to a variety of causes and occurs when the extent of hepatocyte death exceeds the liver's regenerative capacity [1]. Currently, liver transplantation is the “Gold Standard” therapy for the disease. However, due to the limited availability of donor organs and rapid progression of the disease, the mortality of ALF remains high [2]. Therefore, it is imperative to develop novel therapeutic reagents for ALF.

 Qinggan Huoxue Recipe is a traditional Chinese medicine prescription which has been used in China for a long time [3]. Previous literature had indicated that it could improve the liver function in alcohol liver disease models [4–6], but the effects of Qinggan Huoxue Recipe on acute liver failure were rarely explored. 

 In one of our previous prospective clinical cohort studies [7], we found that high-dose Qinggan Huoxue Recipe could significantly improve liver function and coagulation function, reduce complications, and reduce mortality in patients with hepatitis B-related acute-on-chronic liver failure. In order to confirm the effects of high-dose Qinggan Huoxue Recipe on acute liver failure and explore the potential mechanism, we had conducted this experiment.

## 2. Methods

### 2.1. Materials and Methods

All specific pathogen-free (SPF) male Wistar rats weighing 150 ± 20 g were purchased from Shanghai Experimental Animal Co., Ltd (Shanghai, China). D-galactosamine (D-GalN), a commonly used liver injury inducing drug [8, 9], was purchased from Hongbang Medical Technology CO., Ltd (Shanghai, China). Stronger Neo-Minophagen C (SNMC), a classic liver protection drug [10], was purchased from Minophagen Pharmaceutical Co., Ltd (Tokyo, Japan), and fixed into a concentration of 1.56 mg/mL with distilled water. Qinggan Huoxue Recipe which was boiled using *Artemisia capillaris*,* Patrinia, Scutellaria baicalensis, Polygonum cuspidatum, rhubarb*, and red *Peony* (2 : 4 : 4 : 4 : 1 : 4) was purchased from Affiliated Hospital of Chengdu University of Traditional Chinese Medicine (Chengdu, China) with a concentration of 2.97 g/mL.

### 2.2. Design of Animal Experiment

70 rats were randomized into four groups: negative control group (Control, 10 rats) fed with distilled water by gavage; model group (Model, 20 rats) fed with distilled water by gavage and injected with D-GalN 1.4 g/kg intraperitoneally three days after the gavage; Stronger Neo-Minophagen C group (SNMC, 20 rats) fed with SNMC 15.6 mg/kg/d by gavage and injected with D-GalN 1.4 g/kg intraperitoneally three days after the gavage; Qinggan Huoxue Recipe group (Experiment, 20 rats) fed with Qinggan Huoxue Recipe 29.7 g/kg/d (which equals 6.25× clinical dose) by gavage and injected with D-GalN 1.4 g/kg intraperitoneally three days after the gavage. The gavage last for 5 days. The dosage of Qinggan Huoxue Recipe used on rats was calculated by the formula Dose_rat_ = Dose_human_  × (habeas index_rat_/habeas index_human_) × (body weight_human_/body weight_rat_) × 2/3 [11]. Based on this formula, the translational coefficient 6.25 was produced. In our previous clinical trial, the dose of Qinggan Huoxue Recipe used on human was 285 g/60 kg/d (4.75 g/kg/d). At last, the dose of 29.7 g/kg/d was achieved through the multiplication of human dose (4.75 g/kg/d) and translational coefficient (6.25). The dose of 29.7 g/kg/d is a relatively very high-dose used in rats compared with the other studies of Qinggan Huoxue Recipe reported [4, 12]. Besides, a dose response study was carried out in preexperiment to confirm the usage of this high-dose (data not shown). 36 hours after the D-GalN injection, 6 mL blood was collected through femoral artery of alive rats for detection of serum alanine aminotransferase (ALT), aspartate aminotransferase (AST), total bilirubin (TBIL), albumin (ALB), cholinesterase (CHE), and prothrombin time (PT). Then the rats were sacrificed and the left lobe of the liver was collected for further studies such as hematoxylin and eosin (HE) staining, proliferating cell nuclear antigen (PCNA) immunohistochemistry assay, and fluorescence quantitative reverse transcription polymerase chain reaction (qRT-PCR).

### 2.3. Serum ALT, AST, TBIL, ALB, CHE, and PT Determination

The serum biochemical parameters ALT, AST, TBIL, ALB, CHE, and PT which closely reflect the liver function [4] were analyzed by the Department of Laboratory Medicine, Affiliated Hospital of Chengdu University of Traditional Chinese Medicine (Chengdu, China).

### 2.4. Pathological Scores

 The liver specimens were fixed, paraffin embedded, and cut into 3- to 5-*μ*m sections. The sections were used for HE and PCNA staining. The method of HE staining has been introduced in the previous literature [13, 14]. All slides were read by three investigators who were blinded to the allocation arm of the animal. They were asked to grade the microscopic injuries seen in the liver using a semiquantitative scoring system [15], with zero indicating no discernable injury and 4 indicating the presence of severe injury. These scores were allocated based on their assessment of the following histological features: cellular oedema, interstitial oedema, neutrophil infiltration, capillary congestion, and structural distortion. For each slide, the average score of at least three observations was considered as the pathological score.

### 2.5. PCNA Immunohistochemistry

The PCNA immunohistochemistry kit was purchased from Boster Bioengineering CO., Ltd (Wuhan, China). Immunohistochemical staining of PCNA was performed according to the manufacturer's instructions. PCNA-positive cells were counted in 5 random visual fields under 40x magnifications for each section, and the number was expressed as the percentage of PCNA positive cells to the total number of cells counted [16]. Sections were examined microscopically for specific staining, and photographs were taken with a digital image-capture system (Olympus CX40, Tokyo, Japan).

### 2.6. Fluorescence Quantitative RT-PCR

The expression of high mobility group box 1 (HMGB1), toll-like receptor 4 (TLR4), nuclear factor kappa B (NF-*κ*B), and Caspase-3 was detected by qRT-PCR. Total RNAs were extracted with Trizol reagent (Invitrogen) and reverse transcribed into cDNA by the ABI Step-One Plus Real-Time PCR System (Applied Biosystem Co., CA, USA). The primer sequences of the above parameters including *β*-actin were listed in Table 1. 

### 2.7. Survival Curves

Another 55 rats were divided into the above four groups with animal numbers of 10, 15, 15, and 15, respectively, for observation of survival. The treatments were the same as mentioned before. The observation was begun with the time of D-GalN injection, while the endpoint was set at 96 hours after the injection. 

### 2.8. Statistical Analysis

The biochemical parameters, pathological scores, PCNA immunohistochemistry, and mRNA expressions were analyzed by one-way ANOVA, followed by the Student's *t*-test. Survival curves were plotted using the Kaplan-Meier method and analyzed applying the Log-rank test. All statistical analyses were performed using the SPSS 17.0 software package. All *P* values were two sided, and *P* < 0.05 was considered as the significant level of difference.

## 3. Results

### 3.1. The Serum ALT, AST, TBIL, ALB, and CHE Levels and PT

**Figure 1:**
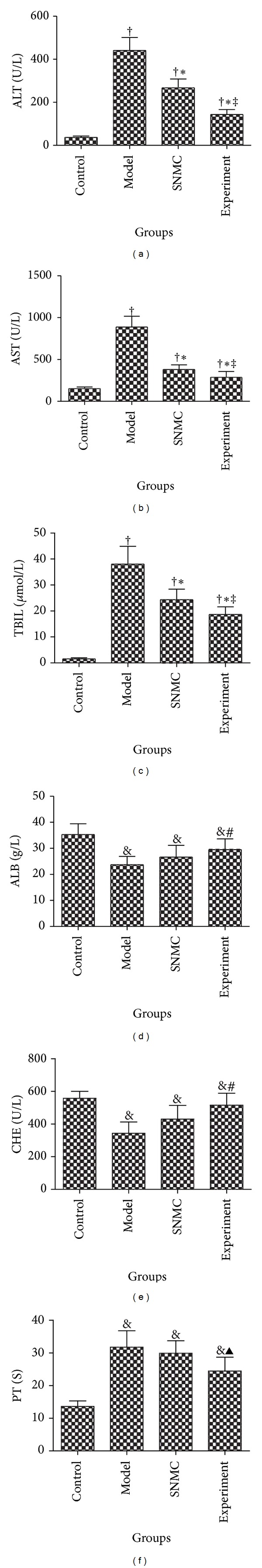
The serum ALT, AST, TBIL, ALB, and CHE levels and PT after treatment. (a) ALT; (b) AST; (c) TBIL; (d) ALB; (e) CHE; (f) PT. Control: negative control group; Model: the group injected with D-GalN; SNMC: the group injected with D-GalN and treated with Stronger Neo-Minophagen C; Experiment: the group injected with D-GalN and treated with Qinggan Huoxue Recipe. For Control, Model, SNMC, and Experiment groups, the ALT levels were 35.15 ± 6.01 U/L, 441.10 ± 60.36 U/L, 267.18 ± 41.45 U/L, and 143.22 ± 22.96 U/L, respectively; the AST levels were 151.61 ± 20.87 U/L, 887.80 ± 128.47 U/L, 380.49 ± 55.38 U/L, and 287.36 ± 68.97 U/L, respectively; the TBIL levels were 1.55 ± 0.43 *μ*mol/L, 38.04 ± 6.84 *μ*mol/L, 24.37 ± 4.03 *μ*mol/L, and 18.65 ± 2.96 *μ*mol/L, respectively; the ALB levels were 35.25 ± 4.19 g/L, 23.67 ± 3.21 g/L, 26.65 ± 4.50 g/L, and 29.46 ± 4.19 g/L, respectively; the CHE levels were 557.40 ± 43.23 U/L, 343.92 ± 68.93 U/L, 430.50 ± 83.53 U/L, and 515.82 ± 73.31 U/L, respectively; and the PT was 13.60 ± 1.73 s, 31.80 ± 5.02 s, 29.93 ± 3.83 s, and 24.46 ± 4.25 s, respectively. For (a), (b), and (c), ^†^ *P* < 0.05 comparing Control group, *P < 0.05 comparing Model group, and ^‡^ *P* < 0.05 comparing SNMC group. For (d), (e), ^&^ *P* < 0.05 comparing Control group and ^#^ *P* < 0.05 comparing Model group. For (f), ^&^ *P* < 0.05 comparing Control group and ^▲^ *P* < 0.05 comparing Model group and SNMC group.

The serum ALT, AST, TBIL, ALB, and CHE levels and PT were shown in Figure 1. From the results, we could find that the ALT, AST, and TBIL were significantly increased, while ALB and CHE were significantly decreased after the injection of D-GalN. PT was remarkably elongated. All these parameters have indicated severe liver damage. However, Stronger Neo-Minophagen C and high-dose Qinggan Huoxue Recipe could improve the liver function by decreasing ALT, AST, TBIL, and PT and increasing ALB, CHE levels. And the effects were more significant in Qinggan Huoxue Recipe group. 

### 3.2. Pathological Scores

**Figure 2:**
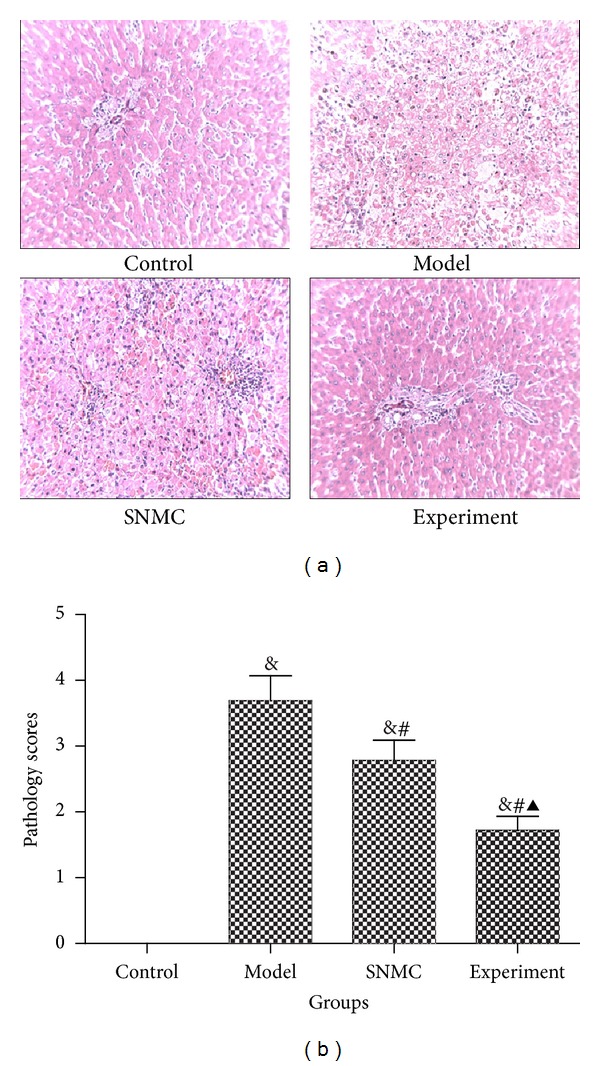
Pathological scores. (a) HE staining; (b) pathological scores. Control: negative control group; Model: the group injected with D-GalN; SNMC: the group injected with D-GalN and treated with Stronger Neo-Minophagen C; Experiment: the group injected with D-GalN and treated with Qinggan Huoxue Recipe. The pathological scores were 0, 3.69 ± 0.38, 2.78 ± 0.31, and 1.72 ± 0.21, respectively, in Control, Model, SNMC, and Experiment groups. ^&^ *P* < 0.05 comparing Control group, ^#^ *P* < 0.05 comparing Model group, and ^▲^ *P* < 0.05 comparing SNMC group.

 As shown in Figure 2, there were massive necroses in the liver tissues of the model group. The necroses were reduced in the SNMC and experiment groups, especially in the experiment group. 

### 3.3. PCNA Immunohistochemistry

**Figure 3:**
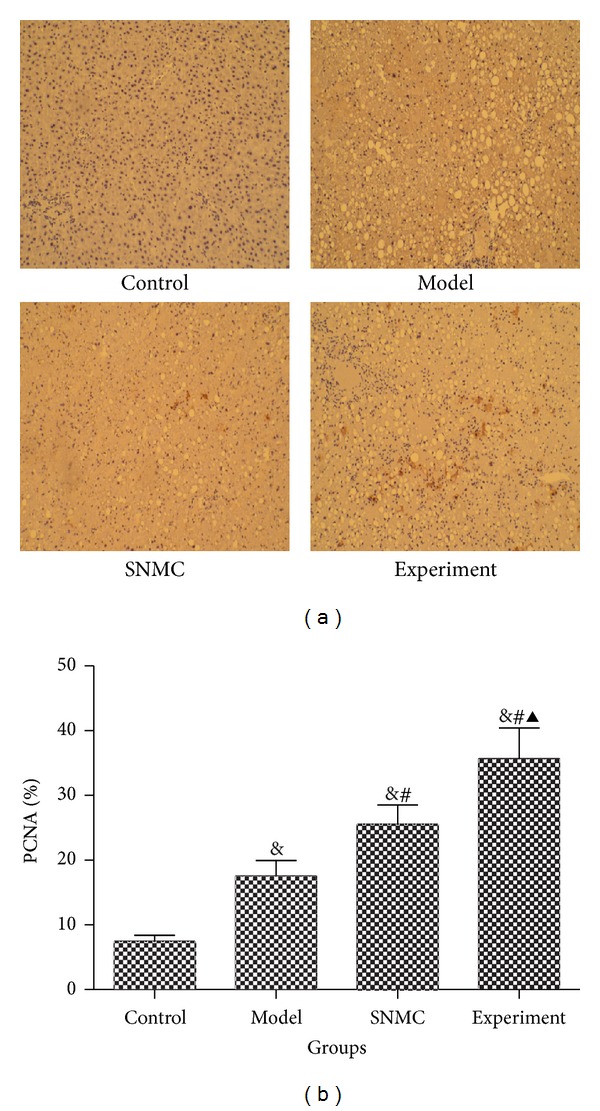
PCNA immunohistochemistry. (a) PCAN immunohistochemistry; (b) histogram of immunohistochemistry. Control: negative control group; Model: the group injected with D-GalN; SNMC: the group injected with D-GalN and treated with Stronger Neo-Minophagen C; Experiment: the group injected with D-GalN and treated with Qinggan Huoxue Recipe. The PCNA positive rates were 7.48 ± 0.90%, 17.55 ± 2.4%, 25.57 ± 2.94%, and 35.68 ± 4.75%, respectively, in Control, Model, SNMC, and Experiment groups. ^&^ *P* < 0.05 comparing Control group, ^#^ *P* < 0.05 comparing Model group, and ^▲^ *P* < 0.05 comparing SNMC group.

The PCNA positive rates were 7.48 ± 0.90%, 17.55 ± 2.4%, 25.57 ± 2.94%, and 35.68 ± 4.75%, respectively, in control, model, SNMC, and experiment groups (Figure 3). The PCNA positive rate of experiment group was significantly larger than the other three groups, indicating high regeneration rates after the treatment of high-dose Qinggan Huoxue Recipe.

### 3.4. The mRNA Expressions

**Figure 4:**
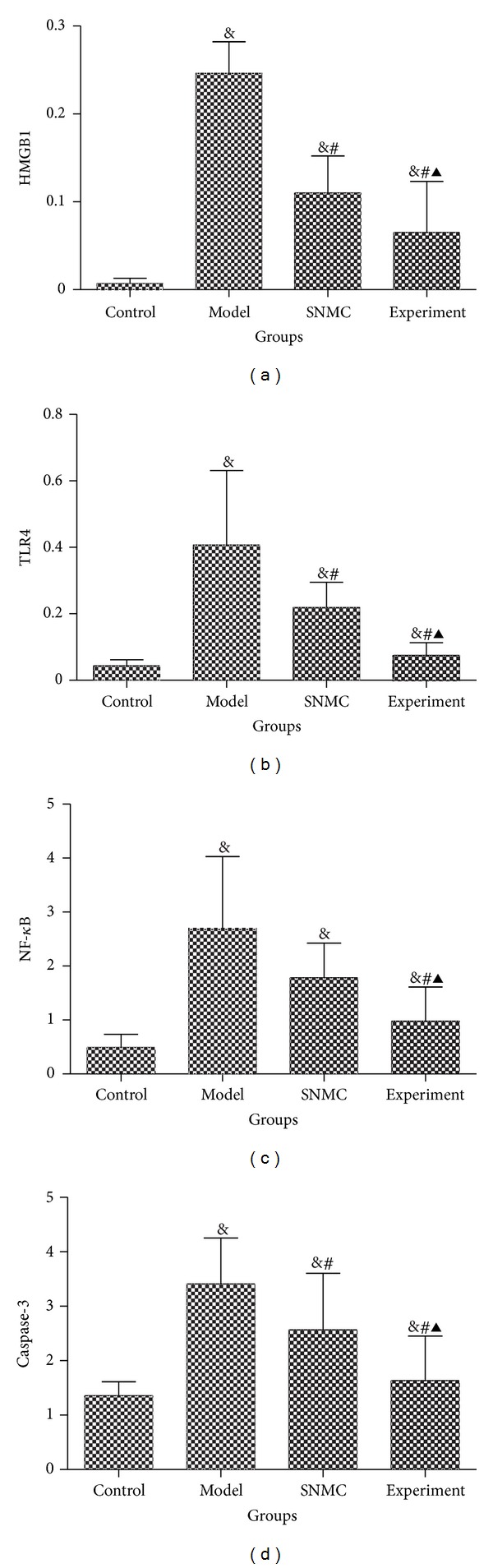
The mRNA expression. (a) HMGB1; (b) TLR4; (c) NF-*κ*B; (d) Caspase-3. Control: negative control group; Model: the group injected with D-GalN; SNMC: the group injected with D-GalN and treated with Stronger Neo-Minophagen C; Experiment: the group injected with D-GalN and treated with Qinggan Huoxue Recipe. For Control, Model, SNMC, and Experiment groups, the HMGB1 mRNA expressions were 0.01 ± 0.01, 0.25 ± 0.04, 0.11 ± 0.04, and 0.07 ± 0.06, respectively; the TLR4 mRNA expressions were 0.04 ± 0.02, 0.41 ± 0.22, 0.22 ± 0.08, and 0.08 ± 0.04, respectively; the NF-*κ*B mRNA expressions were 0.49 ± 0.25, 2.68 ± 1.35, 1.78 ± 0.64, and 0.98 ± 0.63, respectively; and the Caspase-3 mRNA expressions were 1.36 ± 0.26, 3.41 ± 0.85, 2.57 ± 1.04, and 1.64 ± 0.81, respectively. ^&^ *P* < 0.05 comparing Control group, ^#^ *P* < 0.05 comparing Model group, and ^▲^ *P* < 0.05 comparing SNMC group.

 As shown in Figure 4, high-dose Qinggan Huoxue Recipe could remarkably decrease the mRNA expressions of HMGB1, TLR4, NF-*κ*B, and Caspase-3, indicating that it could decrease inflammatory reaction and apoptosis of liver tissues. 

### 3.5. Survival Curves

**Figure 5:**
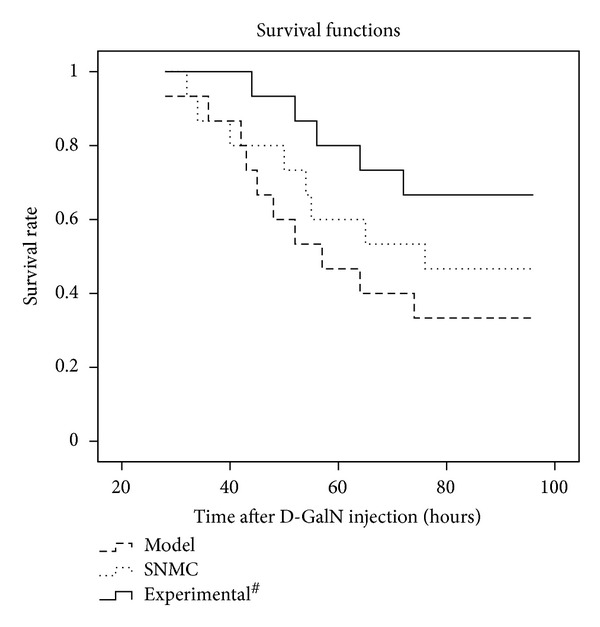
The survival curve. Model: the group injected with D-GalN; SNMC: the group injected with D-GalN and treated with Stronger Neo-Minophagen C; Experiment: the group injected with D-GalN and treated with Qinggan Huoxue Recipe. ^#^ *P* < 0.05 comparing Model group.

 We excluded the control group from survival curve because there was no rat died at the endpoint. From Figure 5, we could found that high-dose Qinggan Huoxue Recipe could improve the survival of acute liver failure model significantly. 

## 4. Discussion 

In this study, the remarkable increased serum ALT, AST, and TBIL levels, elongated PT, decreased serum ALB, CHE levels, increased pathological scores, and rapid death had confirmed that acute liver failure models were successfully built by D-GalN injection. SNMC and high-dose Qinggan Huoxue Recipe could both ameliorate liver function and increase survival times; however, high-dose Qinggan Huoxue Recipe had significant stronger effects. 

The Qinggan Huoxue Recipe used in this study has been practiced for many years in the Affiliated Hospital of Chengdu University of Traditional Chinese Medicine and was found to be very effective in relieving hepatic complications. However, the protective effects of Qinggan Huoxue Recipe on acute liver failure patients were unsatisfactory when used in general dose. So we enhanced the dosage of Qinggan Huoxue Recipe in acute liver failure patients gradually, and we found that liver functions were remarkably improved without discovering any drug-related side effects. In order to confirm the protective effects of high-dose Qinggan Huoxue Recipe on acute liver failure, we had carried out our previous prospective clinical trial [7]. For the Chinese medicine included in this recipe, *Artemisia capillaris* could ameliorate the hydrophilic bile acids-induced hepatic injury which is probably related to a reduced oxidant stress and degree of hepatic fibrosis [17]. *Patrinia* could inhibit the biomarkers related to inflammation through the blocking of NF-*κ*B activation and potentiate anti-inflammatory effects [18]. *Scutellaria baicalensis* could inhibit cyclooxygenase-2 overexpression and therefore alleviates cantharidin-induced rat hemorrhagic cystitis [19]. *Polygonum cuspidatum* was found to be an effective hepatoprotective agent and a promising candidate for the treatment of oxidative stress- and inflammation-related diseases [20, 21]. *Rhubarb* was found to have antioxidant, antiplatelet, and anticoagulant activities and could be used to treat experimental jaundice in rats [22]. Red *Peony* could inhibit inflammation and scavengs free radicals and was found to be effective in treating severe acute pancreatitis [23, 24]. Qinggan Huoxue Recipe has combined these Chinese herb medicines following the principle of clearing heat and resolving stasis. Finally, our previous clinical trial proved that high-dose Qinggan Huoxue Recipe could significantly improve hepatic function in acute liver failure [7], but the potential mechanism is still not for sure. 

HMGB1, a highly conserved, ubiquitous protein that presents in the nuclei and cytoplasm of nearly all cell types, is a necessary and sufficient mediator of inflammation during sterile- and infection-associated responses [25]. Most cells constitutively express HMGB1 and release it on injury or death [26]. It has been suggested that HMGB-1 itself can signal through receptor for advanced glycation end products (RAGEs) and through the toll-like receptors TLR2, TLR4, and TLR9. Activation of these receptors results ultimately in the activation of nuclear factor-kappa B (NF-*κ*B), inducing the upregulation of leukocyte adhesion molecules, production of proinflammatory cytokines, and angiogenic factors in both hematopoietic and endothelial cells, thereby promoting inflammation [27]. Although HMGB1 exerts its cellular and biologic inflammatory responses by binding to three members of TLRs family, namely, TLR2, TLR4, and TLR9, as well as RAGE, TLR4 is the primary receptor of endogenous HMGB1 in mediating cytokine release and tissue damage in various conditions, such as ischemia/reperfusion injury, hemorrhage, and trauma, and this mechanism of injury is attenuated or prevented by deficiency in TLR4 [28]. So we had detected the mRNA expressions of HMGB1, TLR4, and NF-*κ*B in this study. We found that the mRNA expressions of HMGB1, TLR4, and NF-*κ*B were remarkably increased in acute liver failure model group. However, they were significantly decreased after the treatment of SNMC or high-dose Qinggan Huoxue Recipe, especially in Qinggan Huoxue Recipe treatment group. Therefore, we think that high-dose Qinggan Huoxue Recipe could improve liver function in acute liver failure by suppressing inflammation, and this effect was most probably mediated by inhibiting HMGB1/TLR4/NF-*κ*B pathway, though further studies were needed to exclude the interactions between HMGB1 with other receptors. Caspases are crucial mediators of programmed cell death (apoptosis). Among them, Caspase-3 frequently activated death protease, catalyzing the specific cleavage of many key cellular proteins [29]. After the activation of NF-*κ*B, the activities of antigen presenting cells could be enhanced, and then cytotoxic T lymphocytes (CTL) were largely activated, which mediated the apoptosis of hepatocytes [30]. So high-dose Qinggan Huoxue Recipe could also improve liver function in acute liver failure by decreasing the activation of NF-*κ*B and therefore decrease the apoptosis of hepatocytes. 

In this study, we had also found that the PCNA positive rates were significantly higher in high-dose Qinggan Huoxue Recipe group, indicating high regeneration rates. However, whether the promotion of regeneration was produced by direct effects of high-dose Qinggan Huoxue Recipe or the subsequent effects of inflammation suppression was unknown. Besides, further studies were needed to explore the relationship of inflammation, apoptosis, and regeneration in the treatment of acute liver failure. 

Although high-dose Qinggan Huoxue Recipe was used in this study, no drug-related side effects were discovered. In our previous prospective clinical study, the incidence rates of adverse events in the treatment group and the control group were 0.00% and 12.50%, respectively, and the difference was statistically significant. No drug-related adverse events were found in blood, urine and stool routine tests, renal function test, and electrocardiography [7]. During this experiment, we also observed the rats' appetite, behavior change, reaction to stimulation, and so on. We found that the appetites and behaviors of rats in acute liver failure model group were decreased evidently. Besides, they had developed a series of symptoms such as urinary incontinence, yellow urine, listlessness, lethargy, irritability, convulsion, and hemorrhage. However, these symptoms were remarkably decreased after the treatment of high-dose Qinggan Huoxue Recipe, and more important, no treatment-related side effects were discovered. 

## 5. Conclusion

High-dose Qinggan Huoxue Recipe could significantly improve liver function and increase survival rates in rats with acute liver failure. These effects were supposed to be mediated by suppressing inflammatory reaction and apoptosis.

## Figures and Tables

**Table 1:** The primer sequences used in fluorescence quantitative RT-PCR.

Parameters	5′ primer sequence	3′primer sequence
HMGB1	5′TGTTCTGAGTACCGCCCAAA3′	5′TTTCGCTGCATCAGGTTTTC3′
TLR4	5′CCAGGAAGGCTTCCACAAGA3′	5′AATTCGACCTGCTGCCTCAG3′
NF-*κ*B	5′GCACGAGGCTCCTTTTCTCAA3′	5′CGTTTTTCTTCAATCCGGTGG3′
Caspase-3	5′ACCGATGTCGATGCAGCTAA3′	5′AGGTCCGTTCGTTCCAAAAA3′
*β*-Actin	5′AAGGAGGCAAAGGACACCAA3′	5′AATGGCCCCCTTCACAGTTA 3′
